# Child Trauma Experiences and Dissociative Symptoms in Women with Eating Disorders: Case-Control Study

**DOI:** 10.3390/children7120274

**Published:** 2020-12-05

**Authors:** María F. Rabito-Alcón, José I. Baile, Johan Vanderlinden

**Affiliations:** 1 Department of Psychology, Open University of Madrid (UDIMA), 28400 Madrid, Spain; ignaciobaile@hotmail.com; 2 Campus Kortenberg, Universitair Psychiatrisch (Centrum KU Leuven), 3070 Kortenberg, Belgium; johan.vanderlinden@upckuleuven.be

**Keywords:** somatoform, psychoform, trauma, dissociation, eating disorders

## Abstract

Background: many people with different diagnoses, including eating disorders, have suffered traumatic experiences in childhood. Method: a case-control study was performed. The objective of this study was to evaluate the presence of child trauma and dissociative symptoms in people with eating disorders and compare the results obtained with a control group. Participants were administered the Mini International Neuropsychiatric Interview (MINI) and the Structured Clinical Interview for Personality Disorders (SCID-II) to confirm diagnostic criteria and explore possible comorbidities. Traumatic experiences in childhood were evaluated with the Child Trauma Questionnaire in its abbreviated version (CTQ-SF), psychoform dissociation was measured with the Scale of Dissociative Experiences (DES-II) and somatoform dissociation with the Somatoform Dissociation Scale (SDQ-20). Results: women with eating disorders reported a greater severity and higher prevalence of child trauma than the control group. Significant differences were found by groups in dissociative symptoms. Conclusions: our results, in a Spanish sample, confirm the findings of previous studies.

## 1. Introduction

Early exposure to traumatic events, such as repeated sexual and physical abuse during childhood, has a profound negative effect on people’s lives [1]. The consequences of undergoing a traumatic event are complex and may affect individuals by increasing the risk for many health conditions as drug abuse, diabetes, cancer, heart and respiratory diseases and obesity among others [2,3,4] as well as the individual’s relationship with their own body [5,6]. In their study on the link between childhood trauma and dissociation, Scheffers et al. (2017) concluded that childhood trauma seriously affected the patient’s relationship with their body (body attitude, body satisfaction and body awareness). Consequently, traumatized people can find it difficult to detect internal sensations and perceptions, and sometimes even deny any somatic awareness [5,6].

Furthermore, memories of traumatic events, which are often related to the body, can lead to the rejection and loss of contact with the body [5,7]. Previous studies have suggested the link between eating disorders and traumatic events during childhood [2,8,9,10,11,12]. Palmisano et al. (2016) carried out a systematic review of studies which tied trauma with obesity and a binge-eating disorder. Out of the studies analyzed, 87% showed that trauma was a risk factor for the development of obesity and binge-eating disorder (BED). In their systematic review, Caslini et al. (2016) concluded that sexual abuse is linked to the development of bulimia nervosa (BN) and BED, but the results for anorexia nervosa (AN) were not conclusive. Another systematic review on neglect during childhood and eating disorders [13] confirmed the high prevalence of emotional and physical neglect in people with eating disorders when compared with the general population.

Other researchers have tried to explore the mediating variables between traumatic events and the development of eating disorders. According to such studies, some of the possible related factors are lack of emotional self-regulation, self-denigration, borderline personality organization and the presence of dissociative symptoms [14,15,16,17]. In this vein, results from previous researchers have suggested that dissociation plays a significant mediating role in cases of childhood trauma and the development of eating disorders [10,18,19]. Such patients might have “learnt” to dissociate and to resort to behavior such as purging or compulsive eating to avoid or escape trauma-related feelings, sensations, memories and cognition. Or, maybe, they are trying to achieve a less attractive body, even an infertile one in AN patients with a history of sexual abuse. Moreover, vomiting is often linked to feelings of disgust and the quest for purity that come as a result of abuse. In many cases, there might also be a need to punish oneself due to self-blame for the abuse [10,14,15,16,17,18,19].

Mental dissociation would thus emerge as a secondary reaction to trauma. On a behavioral level, this covers numbness, freezing, avoidance, submission and detachment to allow for a state of energy conservation [14]. Some authors have argued that sexual abuse during childhood could trigger a type of dissociative coping mechanism, which results in low self-esteem, poor regulation of emotions and, in some patients, feeling as if life is out of their control. This leads to the need to exert control through disordered self-starvation behaviors [12,14]. Some researchers found a positive relation between early traumatic experiences, dissociation and binge eating behaviors [20,21,22].

Nijenhuis (2000) wrote that “somatoform dissociation” is the result of sexual abuse during childhood. It is defined as a lack of integration of sensorimotor experiences and the mental functions and reactions of the individual. Somatoform dissociation is a somatic manifestation of dissociation, including somatic symptoms which cannot be explained by a medical condition (anesthesia or analgesia, pain, mobility loss...) [23,24]. Researchers on this topic [11,23,25] concluded that psychoform and somatoform dissociation are strongly correlated with early trauma. Some authors found a positive relation between somatoform dissociation and eating disorders [26], and purging [27]. Psychoform dissociation includes amnesia, depersonalization, derealization and identity alteration and confusion in response to traumatic events, especially those which involve physical contact such as physical or sexual abuse [23,24].

Previous studies [11,19,23] have demonstrated this link between traumatic experiences, dissociative symptoms and eating disorders, showing that both past traumatic events and dissociation play a significant role in the etiology and development of eating disorders. In Belgium, Sweden, Italy and Australia [8,11,19,28,29], similar studies have been developed which confirm the link between traumatic events during childhood, dissociation and eating disorders. However, all of these papers concluded that it was necessary to continue research on this topic due to the potential implications for treating people with eating disorders in the future and due to the inconclusive results of other studies regarding the link between these variables [5,9].

In Spain, there is only one published work on the topic [30]: a doctoral thesis. The subject of this paper was to study the link between eating disorders and dissociative disorders, connecting these to the possibility of having suffered early sexual abuse. However, it does not consider other types of abuse, such as physical abuse, emotional abuse, emotional neglect and physical neglect. These researchers concluded that the sample of people with eating disorders presented with higher comorbidity with dissociative disorders than the control group. They also found that BN sufferers had a greater history of sexual abuse than other subtypes of eating disorders included in the study.

A review of the published works on the topic seems to show a connection between trauma, dissociation and eating disorders, but this relationship is not yet clear [10,18,19]. Thus, the objective of our study was to examine the link between (psychoform and somatoform) dissociative symptoms and childhood trauma (sexual abuse, emotional abuse, physical abuse, emotional neglect and physical neglect) in Spanish patients with eating disorders and to compare the results with a Spanish control group.

The following hypotheses will be used in this study:

1. We expected greater (somatoform and psychoform) dissociation in the group of participants with eating disorders than in the control group;
2. We expected greater dissociation amongst the compulsive eating disorder subtypes than amongst the restrictive types;
3. We expected a greater severity/prevalence of traumatic events in the group of participants with eating disorders than in the control group;
4. We expected a greater severity/prevalence of traumatic events amongst the compulsive eating disorder subtypes than amongst the restrictive types.

## 2. Method

### 2.1. Participants

This is a case-control study. We recruited 22 patients: Spanish women from various hospitals and psychological centers who were diagnosed with eating disorders in line with DSM-IV-TR (Diagnostic and Statistical Manual of Mental Disorders) [31] or DSM-5 [32] criteria. In our sample, 68.18% of patients were diagnosed with AN, 13.6% with BN, 9.09% with BED and 9.09% were diagnosed with an eating disorder not otherwise specified (EDNOS). The sample of the group of participants with eating disorders was reflected in the age and gender in the control group, which was made up of 21 women from the general population. Due to the scarce number of men diagnosed with eating disorders in the participating centers, we decided to only include women in this study. Each participant voluntarily consented to take part in the study, after being informed of its objectives. 

All the participants signed informed consent forms. The eligibility criteria for the group of participants with eating disorders were: women over the age of 18 with a current diagnosis of an eating disorder. For the control group, the eligibility criteria were: women over the age of 18 with no known psychiatric diagnosis before inclusion in the study. The study was approved by the Committee on Ethics, Research and Teaching of Madrid Open University meeting the declaration of Helsinki.

### 2.2. Procedure

Each participant in the group with eating disorders had been previously diagnosed by their psychiatrist. To confirm the diagnostic criteria, participants took the Mini International Neuropsychiatric Interview (MINI). We collected sociodemographic data in a data set elaborated for the study. The variables included were age, gender, expected weight, actual weight, expected height, actual height, marital status, eating disorder diagnosis, age of onset of the disorder and the duration of the disorder in years. 

### 2.3. Measuring Instruments

We used the following questionnaires: Clinical Global Impressions severity scale (CGI-S), Mini International Neuropsychiatric Interview (MINI), Structured Clinical Interview for Personality Disorders (SCID II), Dissociative Experiences Scale (DES-II), Somatoform Dissociation Questionnaire (SDQ 20) and the Childhood Trauma Questionnaire (CTQ).

CGI-S [33] is a scale which evaluates the clinical global impression of the severity of the patient’s illness at the time of assessment. There are eight items which range from 0 (“not evaluated”) to 7 (“amongst the most extremely ill patients”). 

MINI [34] is a short structured diagnostic interview. It explores the main diagnoses of DSM-IV Axis I and ICD 10 (International Classification of Diseases) for detection and diagnosis purposes. The MINI is divided into 16 modules, identified by letters, which each correspond to a diagnostic category. At the beginning of each module (except the module on psychotic disorders), one or several ‘filter’ questions are set out in a grey box. These correspond to the main diagnostic criteria of the disorder. At the end of each module, one or several diagnostic check boxes allow the clinic to indicate whether the patient meets the diagnostic criteria. The questions asked throughout the test were closed and must be answered by the interviewer, who responds regarding the presence or absence of the symptom. For this study, we used the Spanish version of the interview [35].

SCID-II [36,37] is an interview which categorically or dimensionally evaluates personality disorders. The questionnaire includes an interview section made up of 119 questions, in addition to others on antisocial disorder. It contains open, closed and yes/no questions to evaluate personality disorders of the DSM-IV Axis II, as well as depressive and passive-aggressive personality disorder. This includes a detection questionnaire of 119 items. For this study, we used the Spanish version of the interview [37].

DES-II [38,39] is a scale with 28 items to be filled out by the patients themselves. It is used to measure a wide range of dissociative experiences, including memory, identity, absorption, depersonalization and derealization problems. Each item is marked in a range of 0–100. Scores over 30 indicate a dissociative disorder. The questionnaire is based on three factors: dissociative amnesia, absorption and depersonalization/derealization. For this study, we used the Spanish version of DES-II, made by Icarán et al. [40,41]. The Spanish version is valid and reliable (Cronbach’s alpha = 0.91) [40,41].

SDQ-20 [42,43] is a questionnaire of 20 items filled in by the patients themselves. It evaluates the presence of somatoform dissociation. These items include symptoms of analgesia, anesthesia, motor problems, altered taste and smell, pain and loss of consciousness. Answers to the items are chosen on a 5-point Likert-type scale, ranging from 1 (“never”) to 5 (“almost always or always”). The sum of these answers equals a total score between 20 and 100. The items include positive and negative dissociative symptoms. For this study, we used the Spanish version of SDQ-20 made by Holm in 2002 and has good psychometric properties [25]. The internal consistency is very high (Cronbach’s alpha = 0.95) [42,43].

CTQ-SF [44] is made up of 28 items, adapted and translated into Spanish by Hernández et al. 2013. The scale measures five types of childhood trauma: emotional abuse during childhood, physical abuse during childhood, sexual abuse during childhood, emotional neglect during childhood and physical neglect during childhood. Answers to the items are chosen on a 5-point Likert-type frequency scale, with scores ranging from 1 (“never”) to 5 (“always”). The CTQ-SF gives both dimensional and categorical levels of abuse. To obtain the categorical level, cut points are used for each of the scales on the questionnaire. To calculate the dimensional level, all of the scores are added together. CTQ-SF is valid and reliable. The Spanish version, used in this study, is valid and internally consistent (Cronbach’s alpha coefficients ranged from 0.66 for emotional neglect to 0.94 for sexual abuse) [45].

### 2.4. Statistical Analysis

For all continuous variables, data was shown as the mean (M) and standard deviation (SD). On the other hand, all categorical variables were expressed as number percentage (%). The data were analyzed with nonparametric tests, because the sample did not fulfil the criteria for normalization. The SPSS program for Windows version 24.0 (IBM Corporation, Armonk, NY, USA) was used to analyze the data. It calculated the descriptive statistics of the variables. The Kruskal–Wallis H test was used to compare the subtypes of eating disorders in the group of participants with a disorder. The Mann–Whitney U test was used to compare the group of participants with eating disorders with the control group. Statistical significance was maintained at *p*-value ≤ 0.050.

## 3. Results

### Socio-Demographic Data

The average age of women in the group of participants with eating disorders was 30.64 (SD = 10.18) with age in the range of 20–54. The average age of women in the control group was 34.29 (SD = 7.72) with an age range between 20 and 49. The average expected weight in the group of participants with eating disorders was 55.86 kg (SD = 22.25), while the average actual weight was 55.8 kg (SD = 26.96). The expected weight in the control group was 61.75 kg (SD = 9.64), while the actual weight was 62.36 kg (SD = 9.83). The averages for expected height and actual height in the group of participants with eating disorders were 164.63 cm (SD = 5.88) and 164.28 cm (SD = 6.43), respectively. The expected height in the control group was 164.38 cm (SD = 5.02), while the actual height was 163.71 cm (SD = 2.62). 

In the group of participants with eating disorders, the average age of onset of the disorder was 21.62 (SD = 10.28), and the average duration of the disorder was 9.19 years (SD = 7.18).

Different subtypes of eating disorders and their correlation with trauma, psychoform and somatoform dissociation.

In order to study the link between different subtypes of eating disorders and trauma severity, we conducted a Kruskal–Wallis H test, which allowed us to establish that the asymptotic significance was 0.530 (χ^2^ = 2.208; *df* = 3; *p* = 0.530). This led us to conclude that there were no statistically significant differences between different subtypes of eating disorders as regards trauma severity (0.530 > 0.005). Using the same test, we analyzed the potential correlation between different subtypes of eating disorders and symptoms of psychoform and somatoform dissociation. Again, no statistically significant differences were found between different subtypes of eating disorders as regards symptoms of psychoform dissociation (χ^2^ = 2.691; *df* = 3; *p* = 0.442), nor as regards symptoms of somatoform dissociation (χ^2^ = 4.064; *df* = 3; *p* = 0.255), and some clear differences were found, however these were not statistically significant. For example, the DES score for the BN group was double that of the AN group (28 versus 14). Therefore, these results do not confirm two of the preliminary hypotheses of this study. On the one hand the one that which put forward the possibility that compulsive subtypes of eating disorders might correlate with higher levels of dissociation. On the other hand, we had expected a higher severity/prevalence of traumatic events in compulsive subtypes of eating disorders. Despite these expectations, the results did not show any statistically significant links between such variables.

Table 1 does, however, show that the averages for all variables were higher in patients with a diagnosis of BN than in patients with a diagnosis of AN. Amongst the former, dissociative symptoms were more prevalent, and there were more cases of traumatic childhood events, or those cases were more severe than amongst the patients with a diagnosis of AN. This was not the case for patients with a diagnosis of BED, who showed lower average values than patients with a diagnosis of AN across all variables, except for amnesia, where they scored the highest of all subtypes of eating disorders. Table 1 provides a summary of all differences found between such different subtypes as regards the following variables: childhood trauma, psychoform dissociation and somatoform dissociation.

Differences between the control group and the group of participants with eating disorders regarding trauma, psychoform dissociation and somatoform dissociation.

In order to test our hypothesis that the group of participants with eating disorders would have more, and more serious, traumatic childhood experiences, we carried out a Mann–Whitney U test compared with the control group. This time, the test concluded that the bilateral asymptotic significance was 0.000, which enabled us to determine that traumatic childhood events were more prevalent/severe in the group of participants with eating disorders than in the control group. We applied the same test across all five different types of abuse. The two groups scored quite differently for emotional abuse, physical abuse, emotional neglect and physical neglect, for which members of the group of participants with eating disorders scored significantly higher. However, the results showed no statistically significant differences between groups regarding sexual abuse. 

We also conducted a set of Mann–Whitney U tests in order to test the hypothesis whereby we had expected higher levels of psychoform and somatoform dissociation in the group of participants with eating disorders. On this occasion, the results showed higher levels of psychoform dissociation in the group of participants with eating disorders, in particular for the depersonalization/derealization factor (0.001 < 0.005). For the rest of psychoform dissociation factors, as well as for the DES-II and SDQ-20 total scores, significant differences were found between the groups, which enabled us to conclude that these hypotheses are confirmed. Table 2 provides a summary of all differences found between the group of participants with eating disorders and the control group as regards the following variables: childhood trauma, psychoform dissociation and somatoform dissociation. 

## 4. Discussion

This study had two main initial purposes. Firstly, it attempted to provide an overview of the differences that may exist regarding childhood trauma, psychoform dissociation and somatoform dissociation in a group of women which had been diagnosed with different subtypes of an eating disorder (subtypes: AN, BN, EDNOS and BED). Secondly, it aimed to compare the results for these variables between a group of women which had been diagnosed with an eating disorder and a control group composed of healthy women with a similar age and the same sex. Our results were similar to those from previous research on this matter [11,29], which showed that trauma was more prevalent/serious amongst participants with eating disorders. Significant differences between groups were found, as regards symptoms of psychoform dissociation and somatoform dissociation. 

Our results are similar from those in the study by Vanderlinden et al. (1996), in which participants with eating disorders showed greater dissociation levels than the individuals in the control group. Incidentally, the results of our research regarding childhood trauma and dissociation of both a psychoform and a somatoform nature for different subtypes of eating disorders were not statistically significant, which was also the case in some previous research in this field of study [8,11]. Despite these similar results, other pieces of research did find statistically significant differences between different subtypes of eating disorders in terms of the prevalence/severity of traumatic events and dissociative symptoms [14,29,46].

An overview of the average scores for all the variables enabled us to notice that average scores tended to be higher in BN cases, a result that is similar to that of a recent study by Palmisano et al. (2018), where there were higher dissociation levels in BN, BED and AN (purging type) sufferers [11]. Carretero et al. (2012) found that trauma exposure had been an on-going occurrence during childhood and most purging subtypes of AN, which might be explained by a more acute need to induce dissociation through impulsive behavior in patients whose condition appears to be more serious. It stands to reason that repeated abuse/trauma will have a greater psychological impact than isolated traumatic abuse/events [47].

Some authors have concluded that post-traumatic stress disorder-related symptoms, such as dissociative symptoms, are frequent in eating disorders, but that there is no correlation between the type or seriousness of the eating disorder and those symptoms [30,48]. However, Vanderlinden and Vandereycken (1999) concluded that, as far as correlation with trauma is concerned, up to 50% of women with an eating disorder suffered sexual abuse in their childhood [49]. This history of sexual abuse was found to be particularly prevalent amongst women with BN. Indeed, sexual abuse in childhood and/or adolescence is a risk factor for the development of eating disorders [30].

It should be noted, however, as a limitation, that traumatic events are not always disclosed by the patient with an eating disorder. Thus, these experiences could be dissociated and may result in false negatives (in cases where patients do not reveal a traumatic event which did, indeed, happen), or false positives (where patients report traumatic events which never happened) [23]. The seriousness of the abuse or abuses reported by the patient, as well as the length of time over which the abuse took place, should also be considered, even though this study did not provide us with any information in that regard. In addition, this study suffered, to some extent, from the limited size of both groups of participants. Many of the results found in this study might change if the number of participants increased including more participants suffering from each specific type of eating disorder, for this would enable researchers to establish relevant comparisons.

In conclusion, traumatic events during childhood as well as psychoform and somatoform dissociative symptoms are all relevant factors in the development of an eating disorder. These factors should be taken into account, and the type of correlation between different variables should be studied in more detail in future research on the topic. Current treatments for eating disorders are largely insufficient in many cases. In this respect, childhood trauma (including sexual, physical and emotional abuse as well as physical and emotional neglect) and dissociation could play a major role in improving success rates for treatments, and as such, they must not be disregarded. As early as 1994, Rosen and Petty outlined the potential correlation between eating disorders and dissociation and called for the inclusion of an educational element to treatment, whereby professionals would teach patients to recognize their own ability to dissociate. Moreover, high levels of dissociation in eating disorders have been proven to hinder the effectiveness of therapies such as cognitive behavioral therapy [18].

This study’s findings, as well as those from previous studies, enable us to better grasp the reason why patients who suffered childhood abuse/trauma and who show dissociation symptoms find it extremely difficult to put a stop to the symptoms of their eating disorder.

In conclusion, we must take into account the fact that eating disorder symptoms may be used by patients as a means to fight the emotional consequences of trauma. Indeed, they are, for many patients, nothing but coping mechanisms aimed at enabling survival.

## Figures and Tables

**Table 1:** Differences between the subtypes of eating disorders as regards the variables: childhood trauma, psychoform dissociation and somatoform dissociation.

Variables	AN	BN	BED	EDNOS	*p*
	Average (SD)	Average (SD)	Average (SD)	Average (SD)	
DES—amnesia	5.75 (5.96)	22.5 (20.11)	26.25 (24.75)	0.62 (0.88)	0.082
DES—absorption	21.68 (13.03)	38.33 (26.16)	12 (8.48)	23 (8.48)	0.459
DES—depersonalization/derealization	12 (14.47)	15 (7.26)	2.5 (3.53)	3.33 (4.72)	0.474
DES—total	14.43 (11.85)	28.02 (17.67)	13.57 (11.11)	9.29 (3.52)	0.442
SDQ-20—total	26.6 (7.92)	39 (19)	21 (1.41)	25.5 (7.77)	0.255
CTQ—emotional abuse	11.4 (5.3)	14.33 (8.14)	6 (0)	13 (2.82)	0.556
CTQ—physical abuse	6.6 (2.13)	12.33 (6.35)	6.5 (2.12)	7 (2.82)	0.563
CTQ—sexual abuse	8.4 (3.56)	8.67 (4.72)	5 (0)	7.5 (2.12)	0.385
CTQ—emotional neglect	11.57 (4.65)	18 (2.64)	7 (0)	17.5 (6.36)	0.059
CTQ—physical neglect	7.64 (3.67)	11.33 (5.86)	6 (1.41)	9.5 (2.12)	0.180
CTQ—total	54.71 (17.14)	69 (24.74)	50 (14.14)	66.5 (12.02)	0.530

Note. AN = anorexia nervosa group, BN = bulimia nervosa group, BED = binge-eating disorder group, EDNOS = eating disorder not otherwise specified, SD = standard deviation, DES = Dissociative Experiences Scale, SDQ-20 = Somatoform Dissociation, CTQ = Child Trauma Questionnaire.

**Table 2:** Differences between the group of participants with eating disorders and the control group as regards the variables: childhood trauma, psychoform dissociation and somatoform dissociation.

Variables	Group with Eating Disorders	Control Group	*p*
	Average (SD)	Average (SD)	
DES—amnesia	9.43 (12.64)	2.32 (4.85)	**0.007**
DES—absorption	23.19 (15.21)	14.04 (8.65)	**0.015**
DES—depersonalization/derealization	10.75 (12.72)	1.98 (7.33)	**0.001**
DES—total	15.74 (12.53)	8.59 (7.65)	**0.026**
SDQ-20—total	27.68 (10.15)	21.73 (2.16)	**0.024**
CTQ—emotional abuse	11.45 (5.44)	6.09 (1.17)	**0.001**
CTQ—physical abuse	7.4 (3.39)	5.42 (0.87)	**0.016**
CTQ—sexual abuse	8.04 (3.44)	6.33 (2.43)	0.063
CTQ—emotional neglect	12.61 (5.23)	8.88 (2.99)	**0.012**
CTQ—physical neglect	8.19 (3,85)	5.38 (0,66)	**0.001**
CTQ—total	57.42 (17.09)	38.42 (6.73)	**0.000**

Note. SD = standard deviation, DES = Dissociative Experiences Scale, SDQ-20 = Significant results marked in boldface, *p* < 0.05. The statistically significant are in boldface.
